# Impact of breed and colostrum replacer quantity on immunoglobulin G kinetics in Holstein and Holstein-Angus crossbred bulls

**DOI:** 10.3168/jdsc.2026-1055

**Published:** 2026-06-11

**Authors:** H.R. McCarthy, A. Kirkup, M. Kovacs, T. Chapelain, J.M. Leão, D.L. Renaud, M.A. Steele

**Affiliations:** 1 Department of Animal Biosciences, University of Guelph, Guelph, ON, Canada N1G 1Y2; 2 Department of Biomedical Sciences, University of Guelph, Guelph, ON, Canada N1G 1Y2; 3 Saskatoon Colostrum Company Ltd., Saskatoon, SK, Canada S7K 6A2; 4 Department of Population Medicine, University of Guelph, Guelph, ON, Canada N1G 1Y2

## Abstract

The objective of this study was to explore the effect of colostrum replacer quantity and breed on serum IgG kinetics in Holstein and Holstein-Angus calves. Holstein (n = 24; 12/treatment) and crossbred (n = 24; 12/treatment) bulls were assigned to 1 of 2 diets for the 0 and 12 h meals: low colostrum replacer (LCR; 2.5 g of IgG/kg birth BW) or high colostrum replacer (HCR; 5 g of IgG/kg of birth BW). Acetaminophen was administered in the 0 h meal to measure abomasal emptying. Calves received milk replacer at 6% BW per meal twice daily from 24 h to d 7. Frequent blood samples were taken to assess serum IgG concentrations, apparent efficiency of absorption (AEA), IgG persistency, and abomasal emptying rate. Serum IgG concentrations were increased in the HCR calves but were not affected by breed. However, there was an interaction of colostrum and breed, where LCR crossbred calves had increased serum IgG concentrations from 4 h to d 7 compared with LCR Holstein calves. Apparent persistency of IgG at 48 h was increased in HCR calves compared with LCR; however, by d 7 apparent persistency of IgG did not differ by treatment. Additionally, apparent persistency of IgG at d 7 was increased in crossbred calves compared with Holsteins, whereas HCR Holstein calves had increased apparent persistency of IgG at 48 h compared with LCR Holstein calves. Calves on the LCR treatment had increased AEA at 24 h compared with HCR, whereas LCR crossbred calves had increased AEA at 12 h compared with LCR Holsteins. Similarly, HCR calves had decreased first-order slow gastric emptying constant (k_SP2_) compared with LCR calves and LCR crossbred calves had increased k_SP2_ compared with LCR Holstein calves. These results suggest that crossbred calves may have a physiological mechanism that allows them to better absorb IgG, particularly when fed low levels of colostrum.

## Graphical Abstract

**Figure:**
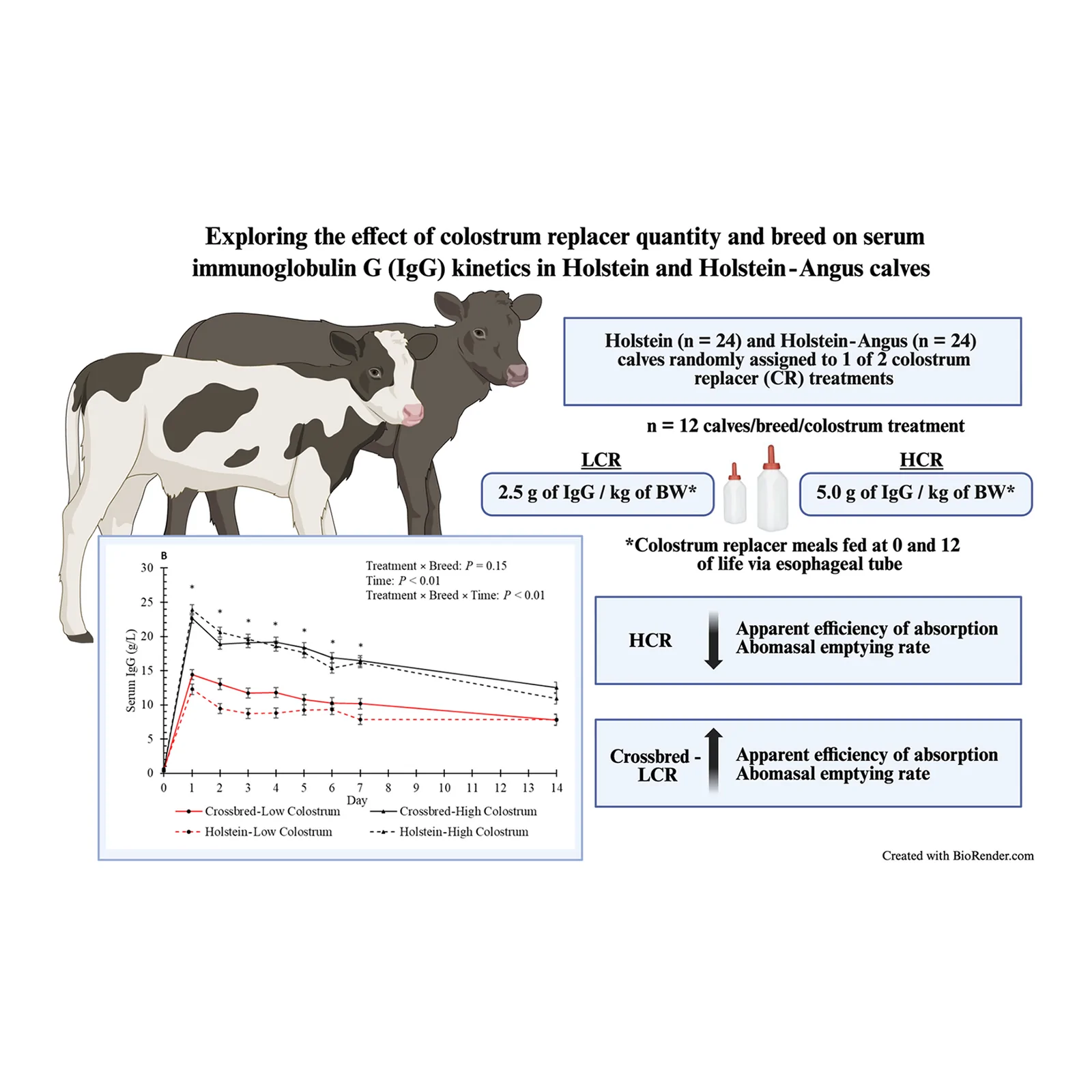

In the past decade, the dairy-beef industry has rapidly expanded, resulting in an increased number of crossbred calves that contribute to global beef production (McWhorter et al., 2020). Reports from calf auctions in Canada show that crossbred calves made up over 60% of calves sold in 2025, a substantial rise from just 7% in 2016 (McCarthy et al., 2026). This reflects the growing use of beef semen in dairy herds and an effort to improve the market value of nonreplacement dairy calves (de Vries et al., 2015; Berry, 2021). Despite this shift, dairy-beef calves are still commonly reared using management protocols developed for nonreplacement dairy calves. This often includes feeding lower quantities of colostrum or colostrum of lower quality (Homerosky et al., 2017; Renaud et al., 2020). As a result, 15% of these calves experience failed transfer of passive immunity (**TPI**), which is associated with increased incidences of diarrhea, bovine respiratory disease, navel infections, and mortality in calves (Johnson et al., 2021; Pereira et al., 2024).

The provision of high-quality colostrum shortly after birth is critical for successful TPI. Neonatal calves are born agammaglobulinemic and immunologically naïve due to the structure of the bovine placenta, which prevents the in utero transfer of immunoglobulins and leaves calves highly vulnerable to infections and disease during the first weeks of life (Duhamel and Osburn, 1984; Godden et al., 2019). Colostrum is rich in IgG and various other bioactive components that support intestinal development, immunity priming, and pathogen defense (Fischer-Tlustos et al., 2021; Morin et al., 2021). Successful TPI through the absorption of IgG from colostrum is essential for providing the calf immune protection and determining the health and survival of the calf (Godden et al., 2019).

Current colostrum feeding recommendations for dairy calves include rapid feeding (within 2 h) of colostrum containing ≥50 g/L IgG (Godden et al., 2019). This contrasts with current recommendations in the beef industry, which suggests feeding colostrum containing ≥100 g/L IgG within the first hours of life (Waldner and Rosengren, 2009). Conventional beef calves regularly achieve serum IgG concentrations greater than 24 g/L, with ∼67% reaching over 24 g/L between d 2 and 8 of life (Waldner and Rosengren, 2009). In contrast, ∼21% of nonreplacement calves, including purebred dairy and crossbred, reach over 24 g/L between d 2 and 8 of life (Kern et al., 2025). These differences in serum IgG concentrations may be the result of different colostrum management or IgG yield of the dam. However, physiological differences in IgG absorption may also partly explain the variation between dairy and beef calves, raising the question of whether crossbred calves absorb IgG differently compared with purebred dairy calves. Therefore, the objective of this study was to explore the effect of colostrum replacer quantity and breed on IgG kinetics in Holstein and crossbred (Holstein-Angus) calves. It was hypothesized that crossbred calves would attain higher serum IgG concentrations than Holstein calves due to enhanced IgG absorptive capacity, and that increasing the quantity of colostrum replacer fed would result in higher serum IgG concentrations due to greater IgG provision.

This study was conducted from July to August 2023 at a commercial dairy farm in Southern Ontario, Canada. All procedures were reviewed and approved by the University of Guelph Animal Care Committee (Animal Use Protocol #4942). The required biological replicate was determined using serum IgG data from a previous study using a similar colostrum feeding protocol (Fischer et al., 2018). Using a mean of 22.3 g/L, a standard deviation of 4.87 g/L, a power of 80%, and an α of 0.05, it was determined that 12 calves per group (48 calves total) were needed to detect a difference of 25% in serum IgG concentration.

Holstein (n = 24) and Holstein-Angus (n = 24) bull calves born from multiparous cows were enrolled in this study. Calves were separated from their dam within 30 min post-parturition and were then weighed using an electronic floor scale (Global Industrial Digital Floor Scale, 1,200 lb [544.3 kg], Global Equipment Company Inc., ON, Canada). All calves received a selenium and vitamin E injection (3.0 mg of sodium selenite, 136 IU of tocopherol acetate E, 1.5 mL s.c./calf; Selon-E, Vetoquinol, QC, Canada) and had their navel dipped with 7% iodine. Calves were blocked by breed and birth BW (≤48 kg or >48 kg) and randomly assigned to one of 2 nutritional treatments (n = 12 calves/breed per treatment) using the RAND function in Microsoft Excel (version 2502, Redmond, WA). Treatments were low colostrum replacer (**LCR**), providing 2.5 g of IgG delivered per kg of birth BW, or high colostrum replacer (**HCR**), providing 5 g of IgG delivered per kg of birth BW. Both treatments used bovine-derived colostrum replacer (**CR**; 27% IgG; Saskatoon Colostrum Company Ltd. [**SCCL**], Saskatoon, SK, Canada) mixed at 27.5% solids using warm water (40°C) and fed at 0 h and 12 h postnatal via esophageal tube. All calves received 2 meals of milk replacer (**MR**; 6% BW/meal, 130 g MR/L, 24% CP, 20% fat, Mapleview Agri Ltd., ON, Canada) at 0700 and 1600 h from d 1 to 14. Between d 2 and 7 of life, calves were transported for 1 h to a calf-rearing facility in Midwestern Ontario for the remainder of the study. All calves received acetaminophen (A5000, Sigma-Aldrich; MilliporeSigma Canada Ltd., ON, Canada) mixed with the 0 h CR meal at 150 mg/kg birth BW^0.75^ as a marker to estimate abomasal emptying rates based on the appearance of acetaminophen in blood after absorption in the small intestine (Schaer et al., 2005). Jugular catheters (16-gauge, 2ʺ [5.1 cm] catheters, Terumo Medical Corp., NJ) were placed at birth before the first colostrum feeding to allow for frequent blood sampling. Serum and plasma blood samples were taken hourly from 0 to 6 h, every 2 h from 6 to 12 h, at 15 h, 18 h, 21 h, 24 h, 36 h, and 48 h post-colostrum feeding to assess serum IgG and plasma acetaminophen concentrations. Blood samples were taken from the jugular catheter using two 10-mL syringes (7 mL of blood per syringe) and a catheter extension kit. After blood collection, 14 mL of saline and 1 mL of heparinized saline were infused into the catheter to replace blood volume and prevent coagulation of blood in the catheter and extension set. Blood was then transferred to plain (serum) and sodium heparin (plasma) 10-mL collection tubes (Vacutainer; Becton, Dickinson, and Co., Franklin Lakes, NJ). Serum samples from d 3, 4, 5, 6, 7, and 14 were taken via jugular venipuncture using 20-gauge, 1ʺ (2.5 cm) Vacutainer needles in 10-mL serum Vacutainer tubes. Serum samples were allowed to clot for 1 h at room temperature before centrifugation. Plasma samples received 3.5 µL of aprotinin and were stored at 4°C until centrifugation. The samples were centrifuged at 2,500 × *g* at 4°C for 15 min. After centrifugation, serum and plasma were aliquoted into triplicate 1.5-mL microcentrifuge tubes and stored at −20°C until analysis. Frozen serum samples were packed with ice packs and shipped overnight to the SCCL Quality Assurance Laboratory to measure serum IgG concentration via radial immunodiffusion analysis (Chelack et al., 1993; Chamorro et al., 2014). Apparent efficiency of absorption (**AEA**) was calculated from 0 to 12 h (**AEA_12h_**) and 0 to 24 h (**AEA_24h_**) using the following formula (Quigley and Drewry, 1998; Cabral et al., 2015):

$$\begin{array}{c} AEA\;\left(\%\right)=\;\\ \frac{birth\;body\;weight\;\left(kg\right)\times 0.09\times serum\;IgG\;\left(\frac{g}{L}24h\right)}{total\;IgG\;fed(g)\;}. \end{array}$$

Area under the curve (**AUC**) was calculated from 0 to 12 h, 0 to 24 h, and 0 to 48 h using the trapezoidal rule to sum the area from serum IgG concentrations at 2 time points (t_1,_ t_2_; Chiou, 1978; Cardoso et al., 2011):

$$AUC\;\left(\frac{g}{L}\times h\right)=\;\frac{\left(IgG_{1}-\;IgG_{2}\right)\times \left(t_{1}-\;t_{2}\right)}{2}.$$

The maximum IgG concentration reached (**C_max_**) and the time to reach the maximum IgG concentration (**T_max_**) were determined. The apparent persistency of IgG in the serum of the calf by 48 h (**IgGPer_48h_**) and d 7 (**IgGPer_d7_**) was calculated using the following formula:

$$IgGPer_{x}\;\left(\%\right)=\left[\;\frac{\;IgG_{x}}{IgG\;C_{max}}\right]\;\times 100.$$

Plasma samples were analyzed for acetaminophen concentration using a commercial ELISA kit (Paracetamol Assay Kit-K8003, Cambridge Life Sciences Ltd., Cambridgeshire, UK). Replicates were accepted when their CV was less than 10%. The inter- and intra-assay CV were 3% and 2%. Abomasal emptying rate was calculated, as described by Stahel et al. (2016), where acetaminophen administered with the colostrum meal serves as a marker for abomasal outflow based on its appearance in plasma following intestinal absorption. The model generates rate constants describing phases of gastric emptying, including the first-order slow gastric emptying constant (**k_SP2_**) and the first-order fast gastric emptying constant (**k_SP3_**), and systemic acetaminophen utilization.

All statistical analyses were performed using SAS software (version 9.4, SAS Institute Inc., Cary, NC). Data were first recorded into Microsoft Excel and manually checked for errors and nonbiologically feasible outliers. Once uploaded into SAS, descriptive statistics were generated, residuals were analyzed using box plots, and normality was assessed using Shapiro–Wilk tests. No outliers were identified in this dataset; therefore, all data points were retained in the analysis. All residuals were assessed for homogeneity and met equal variance assumption. For models using repeated measures, the covariance structure was selected based on the model with the lowest Akaike's information criterion. Statistical differences were determined at *P* ≤ 0.05.

A total of 48 bull calves were enrolled in this study, weighing 47.1 ± 0.64 (mean ± SEM) kg at birth. Baseline serum IgG, before CR feeding, was 0.5 ± 0.05 g/L. There were no differences (*P* > 0.51) between breeds or treatments upon enrollment. Average volume of colostrum consumed was 3.2 ± 0.06 L and 6.3 ± 0.12 L for the LCR and HCR treatments, respectively. Average IgG consumed was 236 ± 4.7 g for LCR calves and 470 ± 8.7 g for HCR calves.

Breed did not directly affect serum IgG concentrations (*P* = 0.17), consistent with previous findings indicating that breed had no effect on serum total protein concentrations upon arrival to a calf-rearing facility (Kovacs et al., 2026). However, a breed by treatment interaction was observed. Crossbred calves receiving the LCR treatment had greater (*P* = 0.05; Figure 1) serum IgG concentrations than LCR Holsteins. Additionally, AUC at 24 and 48 h was greater (*P* < 0.05; Table 1) in LCR crossbreds compared with HCR crossbreds. Crossbred calves also exhibited greater IgG persistency at d 7 (*P* = 0.05; Table 1), whereas HCR Holsteins had greater IgG persistency at 48 h compared with LCR Holsteins (*P* = 0.04; Table 1). These findings may suggest that crossbred calves are better able to maintain IgG concentrations when colostral IgG supply is limited, potentially reflecting differences in physiological efficiency between breeds. However, the mechanisms driving these responses require further investigation.

**Figure 1:**
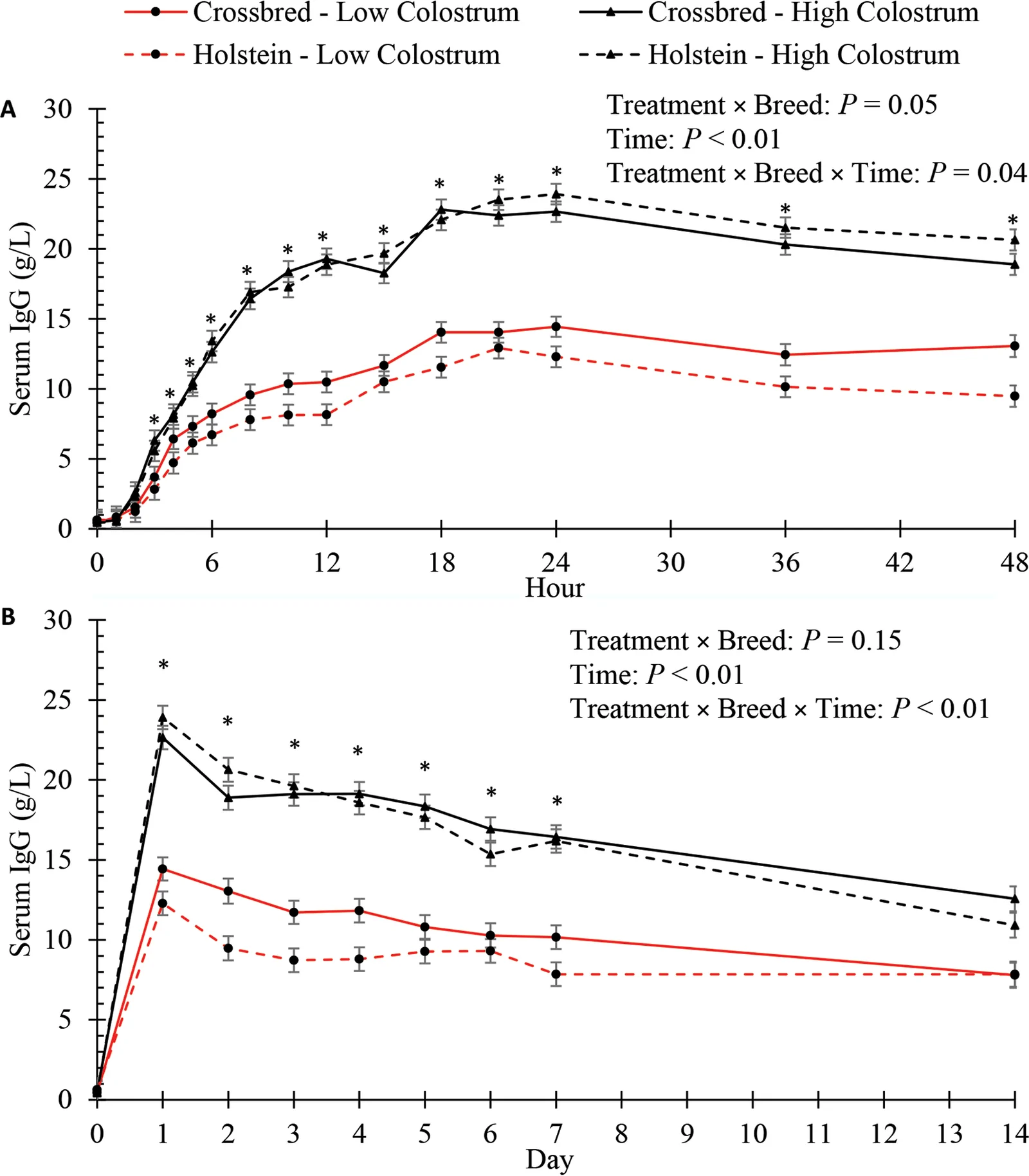
Serum IgG concentrations of Holstein (n = 24) and Holstein-Angus crossbred (n = 24) bulls fed a high (5.0 g of IgG per kg of birth BW) or a low (2.5 g of IgG per kg of birth BW) colostrum replacer treatment at 0 and 12 h after birth from (A) 0 to 48 h and (B) 0 to 14 d. *Denotes significant differences (treatment × breed × time; *P* ≤ 0.05). Error bars denote SEM.

**Table 1:** Serum IgG and gastric emptying characteristics of Holstein (n = 24) and Holstein-Angus crossbred (n = 24) bulls fed a high (HCR; 5.0 g of IgG per kg of birth BW) or a low (LCR; 2.5 g of IgG per kg of birth BW) colostrum replacer treatment at 0 and 12 h after birth

Parameter^1^	Crossbred-HCR	Holstein-HCR	Crossbred-LCR	Holstein-LCR	SE	*P*-value		
						Breed	Treatment	Breed × treatment
IgG								
C_max_ (g/L)	23.4	24.5	16.1	16.3	1.11	0.56	<0.01	0.72
T_max_ (h)	19.5	22.8	23.0	39.3	6.50	0.14	0.13	0.32
AEA_12h_ (%)	34.7^ab^	34.0^ab^	37.7^b^	29.3^a^	1.51	<0.01	0.60	0.02
AEA_24h_ (%)	20.4	21.5	26.6	22.1	1.55	0.29	0.03	0.08
AUC_12h_ (g/L × h)	191.9	191.9	115.4	93.6	5.91	0.07	<0.01	0.07
AUC_24h_ (g/L × h)	646.5^a^	666.5^a^	412.9^b^	335.7^c^	24.27	0.24	<0.01	0.05
AUC_48h_ (g/L × h)	1,341.5^a^	1,403.4^a^	847.9^b^	675.0^c^	50.53	0.28	<0.01	0.02
IgGPer_48h_ (%)	80.5^ab^	84.3^a^	78.2^ab^	70.5^b^	2.64	0.47	<0.01	0.04
IgGPer_d7_ (%)	70.3	65.8	68.0	59.7	3.19	0.05	0.19	0.56
Abomasal emptying								
k_SP2_ (min^−1^ × 10^−3^)	0.32^a^	0.43^a^	0.98^b^	0.43^a^	0.115	0.07	0.01	0.01
k_SP3_ (min^−1^ × 10^−3^)	1.2	1.3	1.6	1.7	0.138	<0.01	0.45	0.97
k_AC UAC_ (min^−1^ × 10^−3^)	0.89	0.97	1.9	1.4	0.29	0.02	0.56	0.39
Time fast (min)	322.5	322.5	257.1	322.5	37.20	0.40	0.40	0.40
Time slow (min)	375.0	352.5	377.1	307.5	50.11	0.67	0.37	0.65
Time off (min)	22.5	45.0	85.7	75.0	27.26	0.10	0.83	0.55

a–c Means within a row with different superscripts differ (*P* ≤ 0.05) when breed × treatment interaction was significant.

1 C_max_ = maximum IgG concentration reached; T_max_ = time to reach maximum IgG concentration; AEA = apparent efficiency of absorption of IgG; AUC = area under the curve; IgGPer = apparent IgG persistency; k_SP2_ = first-order slow gastric emptying constant; k_SP3_ = first-order fast gastric emptying constant; k_AC UAC_ = first-order acetaminophen utilization rate constant.

Breed differences were more apparent in measures related to IgG absorption efficiency and gastric emptying. Crossbred calves had greater AEA at 12 h (*P* < 0.01; Table 1), and there was a breed by treatment interaction where LCR crossbreds had greater AEA_12h_ compared with LCR Holsteins (*P* = 0.02). The increased AEA in crossbred calves aligns with the greater serum IgG concentrations observed in the LCR treatment group. This suggests that crossbred calves may have a greater capacity to absorb IgG when colostral supply is limited, which could contribute to improved passive transfer under suboptimal feeding conditions. The mechanisms regulating IgG transport across the neonatal intestinal epithelium remain incompletely understood (Zhu et al., 2020). One potential explanation is variation in receptor-mediated transcytosis involving the neonatal Fc receptor (FcRn), which facilitates IgG transport across epithelial barriers and protects IgG from intracellular degradation (Pyzik et al., 2019). Differences in FcRn expression or activity between breeds could potentially influence IgG uptake efficiency, although this has not been evaluated in neonatal calves.

Crossbred calves also exhibited altered gastric emptying parameters relative to Holsteins. Specifically, crossbreds had lower k_SP3_ values and greater first-order acetaminophen utilization rate constants (k_AC UAC_; *P* < 0.02; Table 1), and crossbreds fed LCR colostrum had greater k_SP2_ compared with LCR Holsteins (*P* < 0.01). Despite these differences in model-derived rate constants, the total minutes of fast, slow, and no gastric emptying did not differ between breeds or treatments (*P* > 0.10). These findings suggest that subtle differences in gastric emptying kinetics may exist between crossbred and Holstein calves, although the biological significance of these differences is uncertain. One possible explanation is variation in gastrointestinal development or digestive physiology between breeds; however, physiological and hormonal differences between neonatal Holsteins and dairy-beef crossbreds have not been well characterized.

The increased k_SP2_ observed in LCR crossbred calves may provide some insight into their improved IgG absorption parameters. As intestinal permeability to IgG declines rapidly after birth (Stott et al., 1979), faster delivery of colostrum components to the small intestine could theoretically support greater IgG uptake. However, this interpretation should be made cautiously, as similar differences were not observed in calves receiving HCR colostrum treatment. Additional research examining digestive physiology and colostrum feeding strategies in dairy-beef crossbreds compared with Holsteins would help clarify whether breed-specific differences in gastric emptying or intestinal absorption contribute to variation in passive transfer.

Feeding larger volumes of colostrum is a well-established strategy to increase passive transfer in neonatal calves (Godden et al., 2019). Consistent with this, calves receiving HCR treatment had greater serum IgG concentrations, C_max_, and AUC (*P* < 0.01; Figure 1, Table 1), despite a decrease in AEA at 24 h (*P* = 0.03; Table 1). Moreover, calves fed the HCR treatment had greater (*P* < 0.01; Table 1) IgG persistency at 48 h. However, feeding level did not affect IgG persistency at d 7, T_max_, or other abomasal emptying characteristics (*P* > 0.13; Table 1). Hare et al. (2020) previously evaluated IgG persistency using datasets from Morin et al. (1997) and Jaster (2005) and reported that greater persistency was associated with feeding high-quality colostrum. Increased IgG persistency may contribute to higher and more sustained serum IgG concentrations during early life, which is important as greater circulating IgG levels are associated with reduced morbidity and mortality in calves (Lombard et al., 2020). Although calf health outcomes were not evaluated in this study, greater early IgG persistency may support improved disease resilience during the preweaning period.

Feeding larger volumes of colostrum also altered abomasal emptying dynamics. Calves receiving the HCR treatment exhibited a reduction in the gastric emptying rate constant k_SP2_ (*P* < 0.03; Table 1), which represents the slower phase of abomasal emptying derived from the acetaminophen appearance model described by Stahel et al. (2016). In this model, abomasal emptying occurs intermittently and can be characterized by periods of slow or fast outflow from the abomasum based on the rate of appearance of acetaminophen in plasma. The reduction in k_SP2_ therefore suggests that larger colostrum meals may prolong the slower phase of abomasal emptying. Similar responses have been reported previously, where larger volumes of milk or colostrum slowed abomasal emptying in neonatal calves (Burgstaller et al., 2017; Saldana et al., 2019). Slower abomasal emptying with larger meals may reflect increased abomasal distension or altered feedback signals regulating pyloric outflow. Although slower emptying could theoretically delay the delivery of IgG to the small intestine, the higher serum IgG concentrations observed in the HCR treatment indicate that overall IgG absorption was not compromised.

Although this study provides insights into the impacts of breed and colostrum quantity on IgG kinetics, the limitations must also be considered. First, this study used a bovine-derived CR delivered via esophageal tube, which may limit the generalizability of these findings for calves fed maternal colostrum through voluntary suckling or bottle feeding. Colostrum replacers are processed and standardized, and therefore, differ from maternal colostrum in composition and bioactive complexity, which may influence gut development and IgG absorption. Consequently, IgG kinetics and related physiological responses may differ in calves managed under typical on-farm conditions. Second, this study was conducted in bull calves from multiparous Holstein and Holstein-Angus dams, and as such, these results should not be broadly generalized to heifer calves, calves from primiparous cows, or other dairy-beef crossbreeding systems and management strategies. Moreover, calf health outcomes were not evaluated due to limited replication, and therefore the biological relevance of the observed differences in IgG kinetics and persistency to disease risk and early life performance remains unclear. Finally, the mechanisms regulating IgG absorption remain incompletely understood, and further work is needed to define the cellular, molecular, and intestinal factors that regulate IgG transfer and support optimization of passive immunity in neonatal calves.

These results suggest that increasing the amount of IgG fed was associated with greater serum IgG concentrations and IgG persistency, along with reduced AEA and abomasal emptying rates, with similar responses observed across breeds. The breed differences seen in IgG concentrations, IgG persistency, AEA, and abomasal emptying rates when fed low levels of CR suggest that crossbred calves may differ physiologically from Holstein calves; however, more research is needed to fully understand differences.
